# Pneumothorax and Pneumomediastinum Secondary to COVID-19 Disease Unrelated to Mechanical Ventilation

**DOI:** 10.1155/2020/6655428

**Published:** 2020-11-23

**Authors:** Lara Tucker, Sachin Patel, Catherine Vatsis, Antonia Poma, Ali Ammar, Wael Nasser, Satyanarayana Mukkera, Mai Vo, Rumi Khan, Steve Carlan

**Affiliations:** ^1^Department of Internal Medicine, Orlando Regional Healthcare, Orlando, FL, USA; ^2^Division of Critical Care Medicine, Orlando Regional Healthcare, Orlando, FL, USA; ^3^Department of Faculty and Academic Affairs, University of Central Florida College of Medicine, Orlando, FL, USA; ^4^Division of Pulmonary Diseases, Orlando Regional Healthcare, Orlando, FL, USA; ^5^Division of Academic Affairs and Research, Orlando Regional Healthcare, Orlando, FL, USA

## Abstract

In the recent worldwide coronavirus 2019 pandemic, a notable rise in pneumomediastinum and pneumothorax complications has been witnessed in numerous mechanically ventilated patients infected with severe acute respiratory syndrome coronavirus 2. Most cases have reported these complications as barotrauma from mechanical ventilation with COVID-19 disease. We aim to report three polymerase chain reaction-confirmed COVID-19 patients who developed pneumomediastinum and pneumothorax unrelated to mechanical ventilation. We originally analyzed 800 patients with COVID-19 disease at Orlando Regional Medical Center from March 1, 2020, to July 31, 2020, of which 12 patients developed pneumomediastinum and pneumothorax in their hospital course. Interestingly, three patients developed pneumomediastinum on chest imaging prior to intubation. We present these three patients, one female and two males, ages of 42, 64, and 65, respectively, who were diagnosed with COVID-19 disease through nasopharyngeal sampling tests with acute respiratory distress syndrome. Spontaneous pneumomediastinum and pneumothorax are potential complications of COVID-19 disease in the lungs unrelated to mechanical ventilation. This is similar to previous outbreaks of severe acute respiratory syndrome (SARS) and Middle East respiratory syndrome (MERS) diseases. Further investigation is needed to define the causality of pneumomediastinum in nonintubated COVID-19 patients to define the incidence of disease.

## 1. Introduction

On February 11, 2020, the World Health Organization announced a new infectious disease outbreak in Wuhan, China, that eventually led to a worldwide pandemic with a novel coronavirus disease, now referred to as coronavirus 2019 (COVID-19) caused by severe acute respiratory syndrome coronavirus 2 (SARS-CoV-2). At the time of writing, the number of COVID-19 cases has reached over 31 million globally. As time has progressed in this pandemic, healthcare practitioners have witnessed the lethality of this virus and its complications. An interesting complication that has been reported in COVID-19 hospitalized patients is that of spontaneous pneumomediastinum and/or pneumothorax. Majority of the literature has cited high rates of patients on mechanical ventilation with such complications, and it has been theorized that the likely cause is from barotrauma during mechanical ventilation [1–3].

Barotrauma is an air leakage due to wall rupture of marginal pulmonary alveoli secondary to high interalveolar pressure caused by factors such as artificial positive pressure ventilation, forceful coughing, vagal maneuvers, or straining. However, previous experience with severe acute respiratory syndrome (SARS), coronavirus, and Middle East respiratory syndrome (MERS) demonstrated that up to 12% of spontaneous pneumomediastinum were unrelated to barotrauma [3]. This leads into question if there is an inherent component of the coronavirus species that attacks normal lung architecture and predisposes to air leak.

An IRB-approved (20.154.09) retrospective chart review was performed for polymerase chain reaction confirming 800 COVID-19 patients at Orlando Regional Medical Center between March 1, 2020, and July 31, 2020. Of these, 12 patients suffered from pneumomediastinum and/or pneumothorax during their hospital course with all but one requiring mechanical ventilation in their hospital course. Our case series seeks to identify an etiology of spontaneous pneumomediastinum caused by direct viral damage from SARS-CoV-2 by reviewing three unique case presentations, imaging, laboratory, and associated histopathological findings.

## 2. Case 1

**Figure 1:**
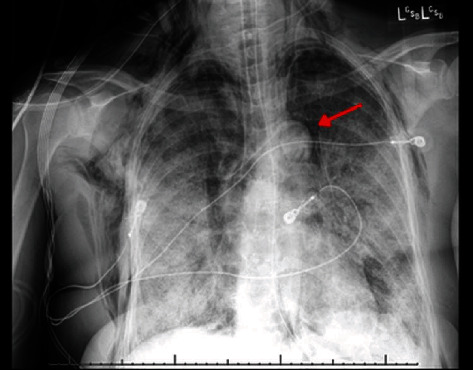
Chest X-ray AP portable shows bilateral progressive infiltrates, basal predominant, pneumomediastinum (red arrow), subcutaneous emphysema, and endotracheal tube in good position.

**Figure 2:**
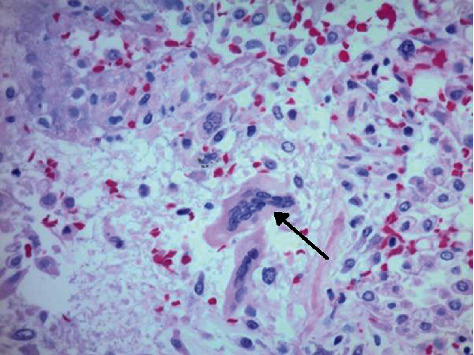
40x magnification, H&E stain, with multinucleated giant cell at arrow.

A 42-year-old African American female with a history of hypertension, sickle cell trait, and herpes simplex virus infection presented with progressive shortness of breath and fatigue for one week. Vital signs were temperature of 37.1°C, 122 beats/min, 33 breaths/min, with blood pressure of 150/99 mmHg, and oxygen saturation of 88% on 15 liters of high-flow nasal oxygen. Physical examination was significant for coarse breath sounds, tachypnea, and accessory muscle use. Laboratory values included complete blood count showing normal white blood cell (WBC) count at 6.2 × 10^3^/*μ*l (normal range of 4.4‐10.5 × 10^3^/*μ*l) with 14% bandemia, elevated C-reactive protein (CRP) at 130.8 mg/L (normal CRP < 9.9 mg/L), elevated D-dimer of 24,895 ng/mL (normal range between 0 and 500 ng/mL FEU) (fibrinogen equivalent units), elevated lactate dehydrogenase (LDH) of 833 U/L (normal range between 140 and 271 U/L), and ferritin of 515.5 ng/mL (normal range between 23.9 and 336.2 ng/mL). Real-time reverse transcriptase polymerase chain reaction (RT-PCR) for COVID-19 was obtained through nasopharyngeal swab and was positive. Chest radiograph (CXR) showed bilateral diffuse ground glass opacities. Patient's overall course deteriorated, and she was placed on a maximum high-flow nasal cannula. A repeat chest radiography showed bilateral worsening infiltrates with pneumomediastinum (Figure 1). Despite aggressive medical management with mechanical ventilator support, empiric antibiotics, remdesivir, paralytics, inhaled epopostenol, diuretics, plasma transfusion, and steroids, the patient succumbed to her disease and died after two weeks of hospitalization. Postmortem results of the lungs (Figure 2) showed diffuse bilateral densely consolidated, congested, and edematous lungs; bilateral peripheral acute pulmonary infarction; and bilateral serosanguineous pleural effusions.

## 3. Case 2

**Figure 3:**
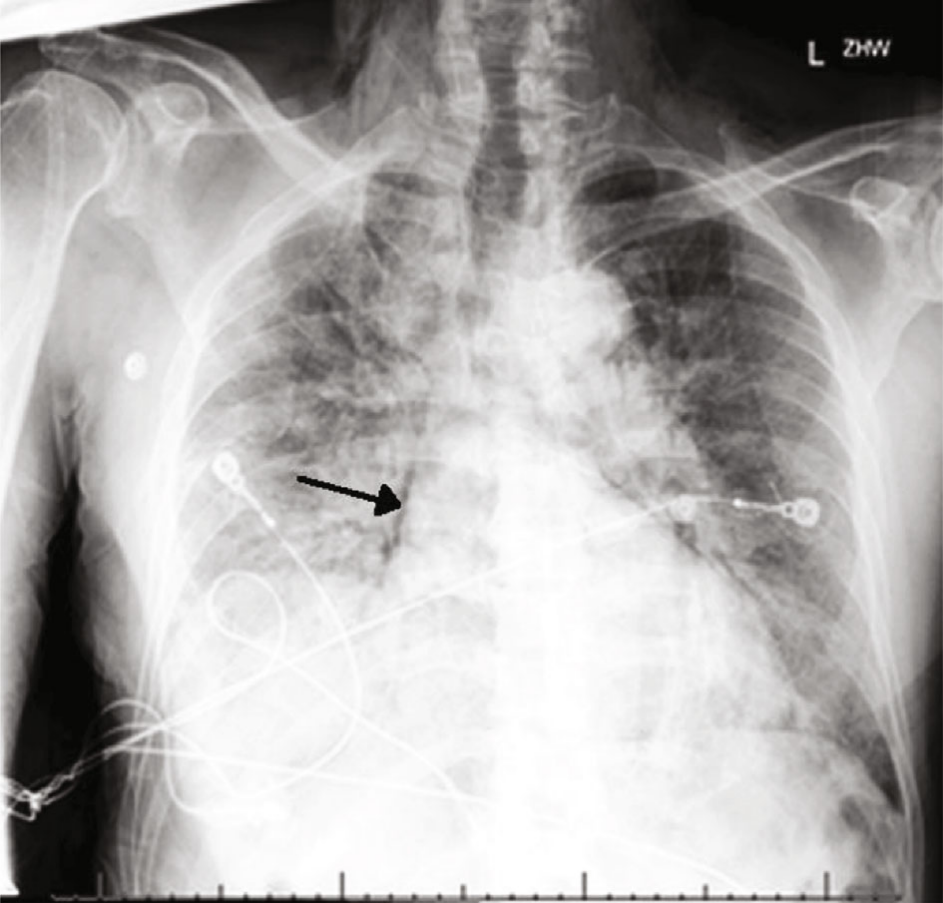
Chest X-ray AP portable with right more than left infiltrates, blunting of right costophrenic angle, and pneumomediastinum noted on right border of heart (arrow).

**Figure 4:**
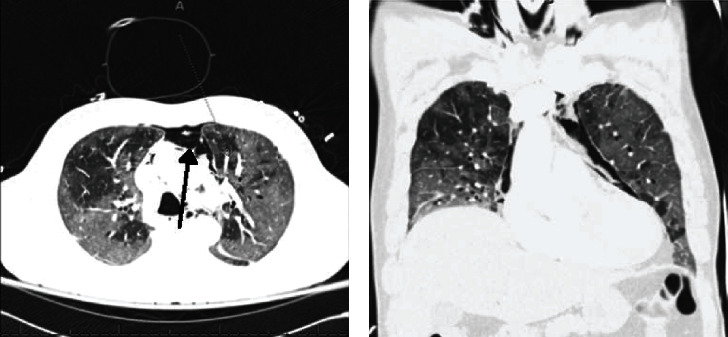
(a) Axial and (b) coronal views of computed tomography of chest. (a) Shows bilateral diffuse ground glass opacities, areas of low attenuation on right lung, anterior pneumomediastinum (arrow). (b) Shows pneumomediastinum and diffuse bilateral infiltrates.

**Figure 5:**
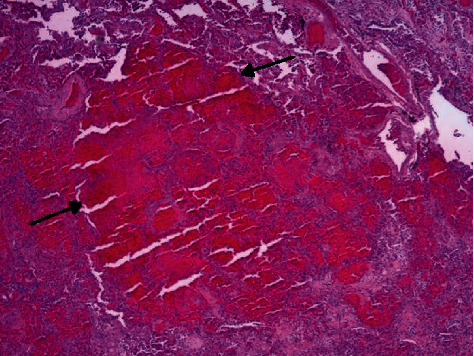
4x magnification—acute hemorrhagic infarction of lung parenchyma (arrows).

A 64-year-old Asian male with a history of active smoking and diabetes mellitus presented with cough, dyspnea, loss of taste sensation, and diarrhea for the past few days. Vital signs were a temperature of 37.2°C, heart rate of 60 beats/min, respiratory rate of 35 breaths/min, blood pressure of 135/91 mmHg, and oxygen saturation of 88% on ambient air. Physical examination was significant for cachexia, tachypnea, and bilateral basal crackles. Laboratory value was significant for a normal WBC count at 7.6 × 10^3^/*μ*l, elevated CRP of 72.3 mg/L, D-dimer of 856 ng/mL, FEU, LDH of 678 U/L, and ferritin of 1261 ng/mL. Patient had a RT-PCR (reverse transcription polymerase chain reaction) for COVID-19 which was positive. CXR (Figure 3) showed diffuse bilateral infiltrates, bibasal atelectasis, and cardiomegaly. He was medically treated with intravenous steroids, antiviral (remdesivir), and convalescent plasma. His oxygen requirements increased to a high-flow nasal cannula of 15 liters combined with nonrebreather mask. Computed tomography (CT) of the chest angiography (Figures 4(a) and 4(b)) showed an acute pulmonary embolus, subsegmental in the right lower lobe and left upper lobe, ground glass opacities in the basal lung field along with pneumomediastinum, pneumothorax, and extensive subcutaneous emphysema. His overall condition continued to decline, eventually requiring mechanical ventilation for his progressive respiratory failure, continuous renal replacement therapy for acute kidney injury, myocarditis, global anoxic brain injury, and died after undergoing cardiorespiratory arrest. The autopsy was significant for mottled myocardium with serous pericardial effusion, bilateral pleural effusions, bilateral edematous lungs, diffuse left lower lobe hemorrhage (Figure 5), and bilateral intravascular thrombi with subcutaneous emphysema of chest wall.

## 4. Case 3

**Figure 6:**
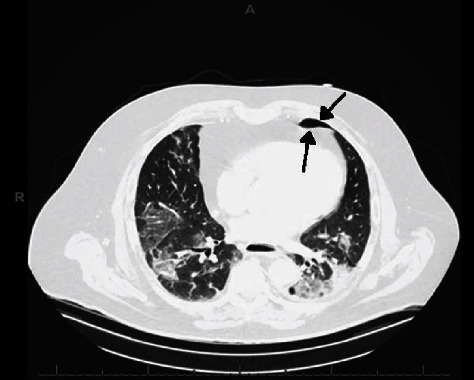
Axial CT chest shows bilateral basal predominant ground glass opacities, patchy left lower lobe consolidation, and left small pneumothorax (black arrows).

A 65-year-old Caucasian male with a history of gastroesophageal reflux disease and morbid obesity presented one month prior with progressive fever, malaise, headache, nausea, and nonproductive cough. He presented again after resolution of the majority of the initial symptoms except for his cough, congestion, and new left pleuritic chest pain radiating to his left arm. Vital signs were temperature of 36.7°C, heart rate of 100 beats/min, respiratory rate of 20 breaths/min, blood pressure of 157/100 mmHg, and oxygen saturation of 97% on room air. Physical examination was only notable for decreased breath sounds bilaterally. Laboratory values included a normal WBC count of 9.8 × 10^3^/*μ*l, but an elevated CRP of 103.8 mg/L, D-dimer of 1129 ng/mL, FEU, LDH of 262 U/L, and no ferritin level was drawn. CXR showed bilateral interstitial infiltrates. CT chest (Figure 6) showed bilateral peripheral predominant ground glass opacities and apical left pneumothorax. During the hospitalization course, the patient was only treated with oxygen support through nasal cannula, two liters per minute, steroids, and antibiotics. Patient was discharged home off supplemental oxygen with follow-up with his outpatient primary care physician.

## 5. Discussion

Pneumomediastinum and pneumothorax can be complications of COVID-19 disease. A literature review reveals multiple reports of air leaks related to barotrauma. We suspect the pathophysiology of COVID-19 disease of the lungs predisposes patients to develop air leak. This was confirmed by all three of our patients presented with combined pneumomediastinum and pneumothorax with elevated inflammatory markers and tachypnea, and two patients passed away due to refractory hypoxemia despite maximum medical support. Interestingly, all of the three patients developed an air leak prior to mechanical ventilation, which highlights that barotrauma is not the only factor associated with pneumomediastinum and pneumothorax in COVID-19 disease.  Table 1 highlights the characteristics of all three patients.

Two of our patients underwent autopsy. Interestingly, both patients' lung weights were increased above normal. Our first patient's histopathology was consistent with organizing diffuse alveolar damage, active infarction, reactive pneumocytes, sparse lymphoplasmocytic inflammation, and areas with fibrin thrombus and multinucleated giant cells. Similarly, our second patient's histopathology specimen of the lungs showed abundant multinucleated giant cells and diffuse alveolar damage with lymphoplasmocytic inflammatory cells but uniquely noted areas of acute hemorrhagic infarction of the lung. We suspect that due to the severe degree of alveolar and interstitial lung architectural damage, this had a significant effect on alveolar ventilation due to hyaline membranes, fibrin thrombi, and infarction. Since both ventilation and perfusion were simultaneously affected, this substantially increased the degree of hypoxemia and shunting, worsened the perfusion and thereby caused pulmonary tissue ischemic with risk for air leak. We believe these pathological findings associated with SARS-CoV-2 infection in the lungs are a plausible explanation for the increased development of pneumomediastinum and pneumothorax [4, 5].

The pathophysiology of COVID-19-induced lung disease with the development of air leak has not been discussed. It is plausible that direct viral infiltration of the lung parenchyma and visceral and parietal pleura caused disruption of parenchymal and pleural integrity or ruptured alveoli leading to subsequent air leak, called the Macklin effect [1, 2]. A cytokine release syndrome (CRS) or overexaggerated immune response could trigger parenchyma and microvascular inflammation with microthrombosis and hypercoagulable states. We suspect that a substantial increase in inflammatory response along with direct viral invasion of the pleura and provoking thromboembolic phenomena are the three major mechanisms or triple hit for the development of air leak [1]. Hence, pneumomediastinum and/or pneumothorax are associated with poor prognoses given the significant inflammatory cascade and viral destruction of lung architecture. In addition, postmortem analysis of our patient did identify a similar lung pathology of severe pulmonary vascular congestion and microvascular thrombosis with hemorrhage to indicate a severe inflammatory insult.

While we highlighted three cases out of 800 patients from March 1 to July 31, 2020, who developed pneumomediastinum and pneumothorax unrelated to mechanical ventilation, we were unable to draw any conclusions of causality. There may have been many other factors that could have provoked the development of spontaneous air leak in these three patients that we did not account for such as the use of bilevel positive pressure ventilation, high-flow nasal cannula at 60 liters per minute which provides an equivalent level of positive end-expiratory pressure of at least four, and anatomic variability due to environmental and genetic causes given size of each patient's lungs, vital capacity, and overall lung compliance. Other contributing variables that may have confounded our results include age, comorbidities, gender, body mass index, previous lung injury, smoking status, structural lung disease, and length of illness. Larger studies are needed to identify patient characteristics that predispose to pneumomediastinum and pneumothorax [2].

In addition, there may have been a selection bias in terms of data collection given individual chart review using the electronic medical records and acquiring all patients with pneumothorax and/or pneumomediastinum with COVID-19 prior to mechanical ventilation. Sampling bias could also have affected our results given the small sample size of patients and was based on International Classification of Diseases-10 (ICD-10) coding for COVID-19. Furthermore, our study was performed at a single institution and may not be generalizable to the entire population. Further studies are needed to identify the incidence of air leak in patients with COVID-19 disease as this may suggest a worse overall prognosis [1, 6].

This case series highlights the pertinent clinical, laboratory, radiographic, and histopathological autopsy features of patients who had pneumomediastinum and pneumothorax unrelated to mechanical ventilation, and these lung findings may associated with worse overall outcomes. More information is needed to answer the clinical questions of placement of chest tubes for pneumothorax to avoid intubation and what level of positive end-expiratory pressure increases the rate of pneumomediastinum and pneumothorax.

## 6. Conclusion

Our cases highlight the notion of direct viral damage with increased inflammatory response as a possible mechanism of pneumomediastinum and pneumothorax in COVID-19 disease.

## Figures and Tables

**Table 1:** Patient characteristics.

Cases	Sex	Age	Race	Lung disease	Days to PM/PTX	Disposition	CRP	Ferritin	D-dimer	Troponin	Steroids	Autopsy findings
Case1	F	42	Black	No	2	Deceased	347	1257	82164	0.03	Yes	Marked diffuse bilateral densely consolidated, congested, and edematous lungs; bilateral possible peripheral acute pulmonary infarctions; bilateral serosanguineous pleural effusions; bilateral peribronchial lymphadenopathy
Case 2	M	64	Eastern Asian	No	4	Deceased	263	832	856	3.72	Yes	Bilateral consolidations with edema, diffuse hemorrhage in the left lower lobe, bilateral fine emphysematous changes especially in the apices, secondary to tertiary intravascular thrombi, and laryngeal and tracheal mucosal congestion
Case 3	M	65	White	No	3	Home	103	n/a	1129	n/a	No	N/A
